# Automatic Gas Control Mode Versus Manual Minimal-flow and Medium-flow Anaesthesia in Breast Surgery: A Comparative Study

**DOI:** 10.4274/TJAR.2025.252143

**Published:** 2025-12-22

**Authors:** Gökhan Çeviker, Özcan Pişkin, Çağdaş Baytar, Rahşan Dilek Okyay, Keziban Bollucuoğlu, Manolya Alkan Canıtez, Bengü Gülhan Aydın, Gamze Küçükosman, Hilal Ayoğlu

**Affiliations:** 1 Zonguldak Bülent Ecevit University Faculty of Medicine Department of Anaesthesiology and Reanimation, Zonguldak, Türkiye

**Keywords:** Consumption, cost analyses, sevoflurane, minimal flow anaesthesia

## Abstract

**Objective:**

This study compared automatic gas control (AGC) mode with manual minimal-flow and manual medium-flow techniques in elective breast surgery, evaluating sevoflurane consumption, cost, hemodynamics, and recovery.

**Methods:**

Following ethics approval, 90 American Society of Anaesthesiologists I-II patients (age 18-65 years) undergoing elective breast surgery were randomized to AGC mode (Group AGC, n = 30), manual minimal-flow control (Group ManCo, n = 30), or manual medium-flow control (Group ModFA, n = 30). All received standard induction after preoxygenation, with maintenance via sevoflurane and remifentanil infusion in a mixture of oxygen and medical air. After reaching a minimum alveolar concentration of 1.0, sevoflurane was adjusted to maintain a bispectral index of 40-60. Mean arterial pressure (MAP), heart rate, peripheral capillary oxygen saturation, bispectral index, inspired sevoflurane fractions and expired sevoflurane fraction, end-tidal carbon dioxide, temperature, and instantaneous sevoflurane consumption were recorded pre-induction and every 15 minutes. Extubation time, recovery time, surgery duration, and total anaesthesia time were documented. Total sevoflurane consumption and cost were calculated postoperatively.

**Results:**

Sevoflurane consumption and related costs were significantly lower in Group AGC versus Groups ManCo and ModFA (both *P* <0.001) and lower in Group ManCo than in Group ModFA (*P* <0.001). MAP and recovery times did not differ significantly among groups (*P* >0.05). Pre-extubation temperature was higher in Group AGC compared to Group ManCo (*P*=0.014) and Group ModFA (*P*=0.002). Extubation time was longer in Group ManCo versus Groups AGC and ModFA (*P* <0.001).

**Conclusion:**

AGC mode significantly reduces sevoflurane consumption and cost compared to both manual minimal-flow and manual medium-flow techniques, without adversely affecting hemodynamics or recovery.

**Main Points**

• Automatic gas control (AGC) mode significantly decreases sevoflurane use, as well as reduces the expense.

• Hemodynamic parameters and recovery times were similar during AGC, manual minimal-flow anaesthesia, and manual moderate-flow anaesthesia.

• AGC mode provides quicker anaesthetic washout and earlier extubation, whereas manual minimal-flow anaesthesia delays extubation.

## Introduction

General anaesthesia is characterised by the reversible loss of consciousness, amnesia, analgesia, and muscle relaxation.^1^ Intravenously administered drugs are used during the induction of general anaesthesia, whereas inhalational agents are delivered using carrier gases, typically oxygen (O_2_) and medical air, for maintenance. The flow rate (L min^-1^) of the carrier gas directly influences the speed and depth of anaesthesia as well as the consumption of the inhalational agent.^2^ Recent technological advances have transformed anaesthesia workstations and monitoring techniques, leading to the development and introduction of low-flow anaesthesia. This method uses rebreathing systems that, after carbon dioxide (CO_2_) absorption, return at least 50% of the exhaled gas mixture to the patient’s lungs. Baker-Simionescu classified fresh gas-flows ≤1 L min^-1^ as low-flow anaesthesia and flows of 250-500 mL min^-1^ as minimal-flow anaesthesia.^2, 3^

Low-flow anaesthesia has substantial economic, environmental, and clinical advantages. The primary advantage of this method is the reduced consumption of carrier medical gases and inhalational anaesthetic agents due to the increased rebreathing fraction. This reduction not only lowers costs but also limits the emission of anaesthetic agents with greenhouse gas properties into the atmosphere, thereby promoting environmental sustainability. Furthermore, this technique enables the maintenance of inspired gas temperature and humidity within physiological ranges, thereby minimizing disruption to airway physiology.^3^

Low-flow anaesthesia can be delivered via manual or automatic gas control (AGC) systems. With manual control, the anaesthetist must continuously monitor the anaesthetic and O_2_ concentrations, and make frequent adjustments to maintain safe and effective anaesthesia. In contrast, AGC systems autonomously regulate fresh gas flow to achieve targeted end-tidal anaesthetic agent (EtAA) and O_2_ concentrations. The AGC mode is an advanced feature that enables fully automated, gas-controlled, minimal-flow anaesthesia.^4, 5^

Although low-flow anaesthesia and AGC have each been investigated previously, few randomized controlled trials have directly compared AGC with manual minimal-flow anaesthesia in terms of both clinical and economic outcomes. To our knowledge, this is the first study to include a three-arm comparison (AGC, manual minimal-flow, and manual medium-flow) in breast surgery, thereby providing not only a direct evaluation of anaesthetic consumption and recovery but also a clinically relevant benchmark for interpreting the advantages of AGC.

The primary outcomes of this study were the effects of AGC and manual minimal-flow techniques on inhalational anaesthetic agent consumption and the cost of breast surgery. Secondary outcomes included assessment and comparison of the effects of these techniques on haemodynamic parameters and postoperative recovery.

## Methods

### Study Design

This prospective, randomized controlled trial was conducted at Zonguldak Bülent Ecevit University Hospital between March 15, 2021, and March 10, 2022, after approval from the Zonguldak Bülent Ecevit University Ethics Committee (approval no.: 2021/05, date: 10.03.2021) and registration at ClinicalTrials.gov (NCT05404269). Written informed consent was obtained from all participants.

### Eligibility Criteria

Women 18-65 years of age with an American Society of Anesthesiologists (ASA) physical status I-II who were scheduled for elective breast surgery under general anaesthesia for ≥1 h were enrolled. Exclusion criteria included coronary artery disease, congestive heart failure, decompensated diabetes mellitus, renal or hepatic insufficiency, chronic obstructive pulmonary disease, opioid sensitivity, malignant hyperthermia, history of tobacco, alcohol, or drug abuse, significant anaemia, sepsis, body mass index >35 kg m^-2^, pregnancy, or lactation, and known allergies to the study medications.

### Randomization

Patients who fulfilled the inclusion criteria were randomly assigned using the sealed envelope method into three groups: AGC [receiving anaesthesia via AGC mode (n = 30)]; ManCo [receiving manual minimal-flow anaesthesia (n = 30)]; and ModFA, the control group receiving manual medium-flow anaesthesia (n = 30).

### Anaesthesia Management

Demographic variables, including age, height, weight, and ASA physical status, were recorded for all patients. All data were monitored and documented by the same investigator (G.Ç.). The same anaesthesia workstation (Maquet Flow-i C20, Getinge AB, Gothenburg, Sweden) was used for every patient. Before each procedure, the anaesthesia circuit was tested for leaks, and the gas monitor was calibrated. Standard safety limits were preset for all alarms, setting the lower threshold for fraction of inspired oxygen (F_i_O_2_) to 30%. A disposable anaesthesia circuit and bacterial filter were used for each patient. Soda lime (KNG-Lime, KNGMED, İzmir, Türkiye) served as the CO_2_ absorbent and was replaced after every use.

Heart rate (HR), non-invasive mean arterial pressure (MAP), peripheral oxygen saturation (SpO_2_), bispectral index (BIS), and train-of-four (ToF) response (IntelliVue MX 550, Philips, Amsterdam, Netherlands) were continuously monitored, and core temperature was measured via a nasopharyngeal probe. The operating room temperature was maintained at 22-24^°^C. Intravenous (IV) access was obtained using an 18-20 gauge cannula in the antecubital vein or dorsum of the hand. 0.9% saline was infused at a rate of 10 mL kg^-1^ h^-1^.

All patients were preoxygenated via face mask with 100% F_i_O_2_ at a rate of 6 L min^-1^ for 3 min using a tidal-volume technique. Anaesthesia was induced with IV lidocaine 1 mg kg^-1^ (Jetmonal 2%, Osel İlaç, İstanbul, Türkiye), propofol 2.5 mg kg^-1^ (propofol 1%, Fresenius Kabi AG, Hamburg, Germany), and fentanyl citrate 1 µg kg^-1^ (Talinat, Vem İlaç, Ankara, Türkiye), followed by rocuronium bromide 0.6 mg kg^-1^ IV (Esmeron, MSD, Hameln, Germany). Endotracheal intubation was performed when the ToF count reached zero. Post-intubation, remifentanil hydrochloride infusion was initiated at 0.05 µg kg^-1^ min^-1^ (Ultiva, GSK Manufacturing S.p.A., Verona, İtaly).

During maintenance, ventilation was set to a tidal volume of 6-8 mL kg^-1^, a respiratory rate of 12-14 breaths min^-1^, an I:E ratio of 1:2, positive end-expiratory pressure 5 cmH_2_O, and end-tidal CO_2_ of 30-40 mmHg. Sevoflurane (Sevorane 100%, AbbVie, Campoverde di Aprilia, Italy) was used in medical air for inhalational maintenance, combined with the remifentanil infusion. Sevoflurane concentration was titrated individually to maintain BIS values between 40 and 60, while neuromuscular management was guided by TOF monitoring (reversal at TOF ≥2; extubation at TOF ratio ≥0.9).

In group AGC, the anaesthesia workstation was preset to AGC mode before intubation (fresh gas flow ≥0.5 L min^-1^; speed of “4”; target EtAA, 1.0 minimum alveolar concentration (MAC); target F_i_O_2_, 40%), and the mode was activated immediately post-intubation.

In the ManCo and ModFA groups, fresh gas flow was manually set to 4 L min^-1^ (approximately 50% O_2_/50% air) with the vaporiser at 2.5% sevoflurane immediately after intubation, and these parameters were maintained for 10-15 min to achieve an end-tidal anaesthetic agent of 1.0 MAC. When the target EtAA concentration was achieved, the flow of fresh gas was adjusted from 0.5 L min^-1^ to 2 L min^-1^.

During maintenance in all groups, fresh gas flow remained constant, sevoflurane vaporiser settings were adjusted to maintain BIS at 40-60, and O_2_ concentration was modified to maintain an F_i_O_2 _of 40%.

During intraoperative monitoring, a MAP <65 mmHg prompted the administration of 5-10 mg IV ephedrine, and an HR <50 beats min^-1^ prompted 0.5 mg IV atropine. If F_i_O_2_ fell to <30%, EtCO_2_ exceeded 45 mmHg, or SpO_2_ dropped to <93%, fresh gas flow was increased to 4 L min^-1^, and the patient was withdrawn from the study.

A multimodal analgesic regimen was administered to control postoperative pain. Approximately 20 min before the conclusion of surgery, patients received IV tramadol 1 mg kg^-1^ (Madol, Koçak Farma, İstanbul, Türkiye), paracetamol 10 mg kg^-1^ (Paracerol, 10 mg mL^-1^ IV vial; Polifarma, Türkiye), and metoclopramide 10 mg (Metpamid, Sifar İlaç, İstanbul, Türkiye) for nausea-and-vomiting prophylaxis.

Approximately 15 minutes before the anticipated end of surgery, anaesthetic discontinuation was initiated. In the AGC group, this was achieved by setting the target EtAA concentration to 0 within the AGC system (speed setting 2), which effectively shut-off agent delivery and triggered automated fresh-gas adjustments for washout. In the ManCo and ModFA groups, the vaporizers were manually turned off at the same time point while the assigned fresh-gas flows were kept constant.

After final skin closure, fresh‐gas flow was increased to 4 L min^-1^ and manual ventilation with 100% O_2_ was applied until spontaneous breathing resumed. Neuromuscular blockade was then reversed using IV atropine (0.02 mg kg^-1^; Atropin Sülfat Ampul, Galen, Türkiye) followed by IV neostigmine (0.05 mg kg^-1^; Neostigmin Ampul, Adeka, Türkiye). Patients were extubated once they achieved a ToF ratio ≥0.9, BIS >80, and adequate spontaneous ventilation. In the post‐anaesthesia care unit, all patients received 4 L min^-1^ O_2_ via a face mask, and those with a VAS score >3 received rescue analgesia with IV tenoxicam (20 mg; Tilcotil, Deva Holding, Türkiye).

Baseline measurements of MAP, HR, and SpO_2_ were recorded before induction. After intubation, the MAP, HR, SpO_2_, BIS, fraction of inspired and expired sevoflurane (F_i_SEVO and F_e_SEVO, respectively), end-tidal carbon dioxide (EtCO_2_), nasopharyngeal core temperature, and instantaneous sevoflurane consumption were recorded at 5, 10, 15, 20, 30, 60, and 90 min.

Extubation time was defined as the interval from vaporiser shut-off, to tracheal extubation, and recovery time as the interval from extubation to achieving a modified Aldrete score ≥9. Surgical duration was measured from the skin incision to the final suture, and the duration of anaesthesia was measured from induction to extubation.

### Statistical Analysis

Sample size was calculated using one-way analysis of variance (ANOVA) of data from a pilot study. After enrolling 10 per group patients, preliminary analysis of sevoflurane consumption indicated an α of 0.05, a β of 0.20 (80% power), and an effect size of 0.33. These parameters indicated that at least 30 patients were required per group.

Statistical analyses were performed using Jamovi version 2.2.5 (Jamovi Project, 2021; https://www.jamovi.org). Continuous variables were evaluated for normality using histograms and the Shapiro-Wilk test. Data conforming to the normal distribution are expressed as mean ± standard deviation, whereas non-normal data are expressed as median (interquartile range, IQR, i.e., 25^th^-75^th^ percentile). For comparisons among ≥3 independent groups, outliers were first identified using boxplots, and variance homogeneity was assessed using Levene’s test. If normality and equal variances were confirmed, one-way ANOVA was performed using Fisher’s F test, when variances were equal, or with Welch’s correction when they were not, followed by Tukey’s or Games-Howell post-hoc tests, each of which includes adjustment for multiple comparisons to control type I error. Non-parametric data or data violating ANOVA assumptions were analyzed using the Kruskal-Wallis test with Dwass-Steel-Critchlow-Fligner post-hoc comparisons, which also incorporate multiplicity adjustment. Categorical variables are expressed as number (n), and percentage, and are compared using Pearson’s chi-squared or Fisher’s exact tests. All statistical tests were two-sided, and differences with *P* <0.05 were considered to be statistically significant

## Results

**Figure 1:**
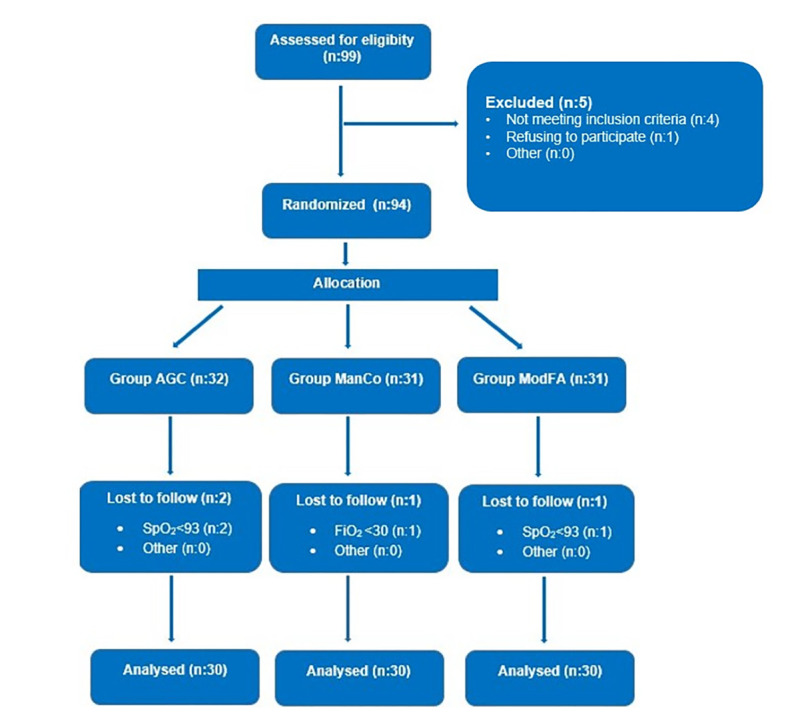
CONSORT flow diagram of the study. AGC, automatic gas control; ManCo, manual minimal-flow control; ModFA, manual medium-flow control; F_i_O_2_, fraction of inspired oxygen; SpO_2_, peripheral oxygen saturation.

A CONSORT flow-diagram illustrating patient enrolment is presented in Figure 1. Ninety patients were included. There were no significant differences among the groups in terms of demographic characteristics or ASA physical status (*P* >0.05) (Table 1).

There were no significant differences in surgical, anaesthetic, or recovery times between the groups (*P* >0.05). However, extubation time differed significantly among the groups (*P* <0.001) (Table 2). Pairwise comparisons demonstrated that extubation times in the ManCo group were significantly longer than those in the AGC and ModFA groups (both *P* <0.001). No significant differences were observed between the AGC and ModFA groups (*P* >0.05).

Sevoflurane consumption differed significantly among the groups (*P* <0.001) (Table 3). Hourly consumption was lower in the AGC group than in both the ManCo and ModFA groups (*P* <0.001 for each comparison) and was also lower in the ManCo group than in the ModFA group (*P* <0.001).

Sevoflurane-associated costs also differed significantly among the groups (*P* <0.001) (Table 3). The costs in the AGC group were lower than those in both the ManCo and ModFA groups (*P* <0.001 each), and the costs in the ManCo group were also significantly lower than those in the ModFA group (*P* <0.001).

**Figure 2:**
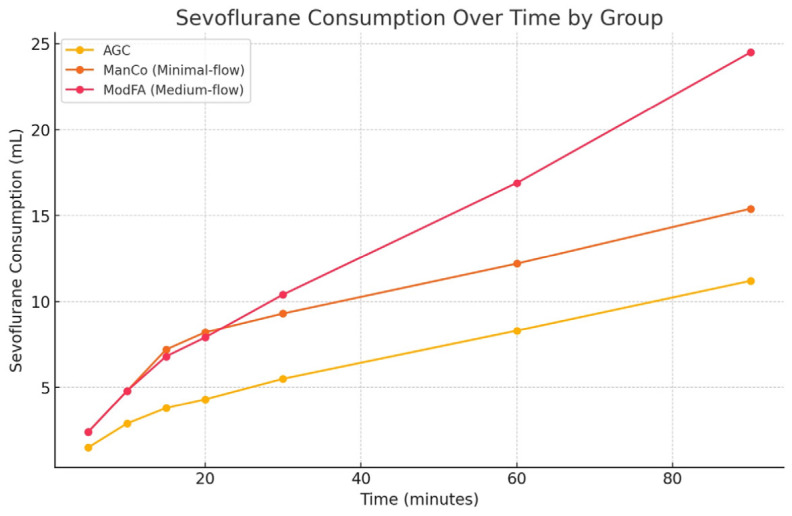
Comparison of time-point sevoflurane consumption values between groups. AGC, automatic gas control; ManCo, manual minimal-flow control; ModFA, manual medium-flow control.

Statistically significant differences were observed among the groups in both instantaneous and total sevoflurane consumption (*P* <0.001) (Figure 2, Table 4). In the post-hoc analyses, no significant difference was observed between the ManCo and ModFA groups in instantaneous sevoflurane consumption at 5, 10, or 20 minutes (*P* >0.05).In contrast, all other pairwise comparisons indicated that consumption in the AGC group was significantly lower than that of both the ManCo and ModFA groups (*P* <0.05), and that consumption in the ManCo group was significantly lower than that of the ModFA group (*P* <0.05).

There were no significant differences among the groups in MAP, SpO_2_, or EtCO_2_ at any time point (*P* >0.05). At the 5^th^ minute after intubation, the mean HR values were 83 beats/min in the AGC group, 86 beats/min in the ManCo group, and 81 beats/min in the ModFA group. Although the overall analysis revealed a statistically significant difference (*P*=0.042), post-hoc comparisons showed no clinically relevant intergroup differences. No other time points demonstrated statistically significant differences (*P *>0.05).

Pairwise comparisons of F_i_SEVO values showed that at 5 and 10 minutes, the AGC group had significantly lower values than both the ManCo and ModFA groups (*P* <0.05). At 15 and 20 minutes, F_i_SEVO values were significantly lower in the AGC group compared with both the ManCo and ModFA groups, and the ManCo group also had significantly lower values than the ModFA group (all *P* <0.05). For F_e_SEVO, values at 5 and 10 minutes were significantly lower in the AGC group compared with both ManCo and ModFA groups (*P *<0.05). At 60 minutes, F_e_SEVO values in the AGC group were significantly lower than in the ModFA group (*P* <0.05), while no significant differences were observed between AGC and ManCo, or between ManCo and ModFA.

There were no statistically significant differences in BIS values among the groups at any recorded time point (*P* >0.05, Table 5).

There were no significant differences in body temperature among the groups at any time point, except immediately before extubation (*P*=0.002). Immediately before extubation, a significant difference in core temperature was observed among the groups (*P*=0.002). Pairwise comparisons showed that the AGC group had significantly higher values compared to both the ManCo and ModFA groups (*P*=0.014 and *P*=0.002, respectively), while no significant difference was found between the ManCo and ModFA groups (*P* >0.05).

During the study, four patients were excluded due to hypoxemia. One patient in the ManCo group was excluded because F_i_O_2_ decreased below 30%. In the AGC group, two patients were excluded due to SpO_2_ dropping below 93%, and in the ModFA group, one patient was excluded for the same reason.

## Discussion

In this randomized controlled study, AGC significantly reduced sevoflurane consumption and costs compared with manual minimal-flow and medium-flow anaesthesia, without compromising hemodynamic stability or recovery profiles. These findings indicate that AGC provides both economic and clinical advantages over conventional techniques. Although the primary objective of our study was to compare AGC with manual minimal-flow anaesthesia, we also included a manual medium-flow group to reflect routine clinical practice and to provide a broader context for interpreting the differences across three distinct flow strategies.

Low-flow anaesthesia is generally not recommended for short procedures, with the literature indicating that procedures lasting <30 min are not suitable for this method. ^6^ In our study, the mean duration of anaesthesia was 132 minutes, confirming that the patient cohort was appropriate for low-flow anaesthesia.

The primary disadvantages of inhalational agents in general anaesthesia are their high cost and the environmental damage caused by their greenhouse-gas emissions.^3, 7^ Modern anaesthesia workstations designed to handle these problems use integrated software to produce accurate gas mixtures and constantly regulate fresh gas flow to maintain the optimal agent concentration.^8^ In our study, we used the Maquet Flow-i Anaesthesia Workstation, which meets all of these monitoring requirements, electronically controls fresh gas supply, and maintains system leaks to 150 mL min^-1^.

Although several volatile agents can be employed in low-flow anaesthesia, sevoflurane is one of the most frequently chosen due to its low blood-gas solubility and its ability to be administered at large concentrations.^9^ Compounds A and B have been found to accumulate during sevoflurane-based low-flow anaesthesia that continues for greater than 5 h; however, this does not appear to have a clinically significant effect on renal or hepatic function.^10^ Considering these risks, we excluded cases exceeding 4 hours from the study and used sevoflurane as the inhalation agent.

Reducing fresh gas flow during low-flow anaesthesia can decrease the delivered O_2_ concentration and increase the risk of hypoxia. To reduce this risk, an inspired O_2_ concentration ≥30% is recommended.^9^ Although hypoxemia was uncommon, four patients had to be excluded for safety reasons (one in ManCo, two in AGC, and one in ModFA). This highlights the importance of continuous oxygenation monitoring, particularly under low- and minimal-flow anaesthesia, where transient fluctuations may pose clinical risks.

Intraoperative awareness during general anaesthesia has been found to occur in approximately 0.1-0.2% of cases.^11^ Because low-flow procedures may increase the risk of awareness, we used BIS monitoring in all patients to determine anaesthetic depth and reduce the potential of intraoperative awareness. During the study, eight patients reported BIS values >60; in this group, the inhaled sevoflurane concentration increased until the BIS decreased to <60. A comparison of BIS values among the groups indicated no statistically significant differences.

Maintaining haemodynamic stability during anaesthesia is crucial for patient safety. Ceylan et al.^12^ found that maintaining a fresh gas flow rate of 1 L min^-1^ under low-flow anaesthesia did not impair haemodynamic parameters. Similarly, Skalec et al.^13^ compared AGC and manual gas control under low-flow anaesthesia and found no significant hemodynamic differences. In our study, no significant intergroup differences in haemodynamic parameters were observed, except for MAP 5 minutes after intubation. At 5 min post-intubation, the HRs were 83, 86, and 81 beats/min in the AGC, ManCo, and ModFA groups, respectively, with no clinically significant intraoperative effects.

The addition of remifentanil infusion to anaesthesia maintenance contributes haemodynamic stability. Remifentanil alone is not associated with hypnosis or loss of consciousness; however, when combined with anaesthetics, it enhances their effects.^14^ In a study comparing minimal-flow anaesthetic efficacy and sevoflurane versus desflurane consumption, using a remifentanil infusion during maintenance was found to significantly improve hemodynamic stability.^15^ In another study comparing AGC mode with manually regulated minimal-flow anaesthesia, remifentanil infusion significantly improved intraoperative analgesia management.^5^ In our study, to support hemodynamic stability, we administered a remifentanil infusion at 0.05 µg kg^-1^ min^-1^ during anaesthesia maintenance.

Several variables affect recovery following general anaesthesia, including the pharmacokinetic characteristics of the inhalational medication and patient ventilatory capacity.^16^ The most commonly used method for assessing recovery is the modified Aldrete score, with a score ≥9 considered to be acceptable.^17^ Our study demonstrated no significant difference in time for achieving a modified Aldrete score ≥9 among the three groups. In a study using sevoflurane for anaesthesia maintenance, the end-tidal and manual control techniques were compared, and the interval from sevoflurane discontinuation to extubation was reported to be similar in both groups.^18^ Similarly, Lortat et al.^19^ compared the AGC and manual control methods in low-flow desflurane anaesthesia and found no significant difference in extubation time. In this study, the interval from vaporiser shut-off to tracheal extubation was defined as “extubation time.” Our results demonstrated that the extubation time was significantly longer in the ManCo group than in both the AGC and ModFA groups, with no significant difference between the AGC and ModFA groups. Although the extubation times varied among the groups, the similarity in recovery duration was likely due to our study design. We believe that the primary cause of the prolonged extubation time in the ManCo group was that, although the vaporiser was turned off approximately 10-15 minutes before the conclusion of surgery, the fresh gas flow was not increased during this interval under the minimal-flow technique. It should also be considered that the longer extubation time in the ManCo group may partly reflect the standardized timing of vaporizer shut-off across groups, in addition to the slower anaesthetic washout under minimal-flow conditions. After vaporizer discontinuation, F_i_SEVO decreases toward zero; however, under minimal-flow conditions (0.5 L min^-1^), the long circuit time constant leads to a slower fall in FeSEVO, thereby prolonging anaesthetic washout. These findings demonstrate the potential advantages of AGC in reducing extubation time.

Hypothermia is a common and serious complication of general anaesthesia. Preservation of heat and humidity is essential not only for maintaining thermoregulatory mechanisms but also for ensuring normal airway physiology.^9^ Bilgi et al.^20^ demonstrated that low-flow desflurane anaesthesia more effectively preserved respiratory function and mucociliary clearance than high-flow administration. All patients in our study were fitted with heat-and-moisture-exchange filters, and core temperature was monitored via a nasopharyngeal probe, with values ranging from 35.7 **^°^**C to 36.8 **^°^**C. Pre-extubation measurements revealed that patients in the AGC group maintained higher temperatures than those in the ManCo and Group 3 cohorts, suggesting that AGC may be more effective in preserving thermal homeostasis.

Low-flow anaesthesia reduces greenhouse gas emissions and consumption of inhalational agents, leading to economic advantages.^21, 22, 23^ In one study, anaesthesia accounted for 1% of total hospital costs. Anaesthetic medications accounted for 5.7% of total pharmaceutical spending, with inhalational agents accounting for 20% of that.^16^ The primary factors affecting the cost of volatile anaesthetic agents include the selling price, potency, administered concentration, and fresh gas flow rate. Among these parameters, only the fresh gas flow rate was clinically variable. Reducing the fresh gas flow rate improves rebreathing, which reduces consumption of the inhalational agent and waste gas emissions. As a result, fresh gas flow becomes the most important predictor of agent cost; more specifically, as flow decreases, so do consumption and cost.^16, 24, 25^ There are several techniques used in low‑flow anaesthesia, two of which are AGC and manual control. With AGC, the device automatically adjusts both the vaporiser and fresh gas flow to reach and maintain the target inhalational agent concentration. In manual control, these adjustments are made by the operator.^26^ Tay et al.^3^ compared end-tidal gas concentration monitoring by AGC versus manual control in approximately 3600 general anaesthesia cases. In the AGC group, hourly inhalational agent consumption was, on average, USD 5 lower, total anaesthesia costs decreased by 27%, and the greenhouse-gas footprint was reduced by 44%. The manual control group demonstrated increased agent consumption, although this difference was not statistically significant. The investigators in that group frequently adjusted the vaporiser and O_2_ settings.^18^ Carette et al.^26^ reported that on the FLOW-i workstation, the AGC mode not only reduces costs, but also reduces the workload of low-flow anaesthesia by substantially decreasing the frequency of vaporiser and oxygen adjustments. Lortat et al.^19^ reported that the use of AGC in desflurane anaesthesia significantly reduced agent consumption. Similarly, in our study, mean sevoflurane consumption was 7.01±1.10 mL h^-1^ in AGC mode, 9.80±1.54 mL h^-1^ in manually controlled minimal-flow, and 15.05±1.28 mL h^-1^ in manually controlled moderate-flow. Similarly, sevoflurane costs were calculated for each mode. They averaged 24.6 Turkish Lira (TL) per hour in the AGC mode, 35.6 TL per hour in manually controlled minimal flow, and 55.7 TL per hour in manually controlled moderate flow. These findings strongly indicate that the AGC mode has a significant advantage in terms of reducing agent usage and associated costs. This significant reduction associated with AGC supported its economic efficacy. The study’s main methodological advantages for an effective cost analysis included comparable demographic characteristics between groups and standardised surgery and anaesthetic durations. Furthermore, by using the same breast surgery process in all cases and a single anaesthesia workstation, the design was improved.

In our study, the number of patients analyzed declined progressively after 70 minutes. However, the advantages of low-flow anaesthesia in terms of inhalational agent use and cost have become apparent with longer procedures. One of the main limitations of this study is that, although our findings support the low-flow technique, understanding its environmental and economic implications requires additional research with longer procedures and larger patient cohorts.

Another limitation was the exclusion of participants with ASA scores ≥III. Additionally, smokers were excluded to maintain group homogeneity. The design of this study improved the internal validity; however, it limited the generalizability of our findings to larger patient groups.

## Conclusion

In this study of patients undergoing breast surgery, we compared minimal-flow anaesthesia procedures using AGC versus manual gas control in terms of inhalational agent use, cost, haemodynamic parameters, and recovery time. Sevoflurane consumption was significantly lower in the AGC group than in the manually controlled minimal-flow and moderate-flow groups. This decrease was also observed in cost analysis, confirming the economic benefits of AGC. Furthermore, there were no statistically significant differences among the groups in haemodynamic parameters or recovery time; this demonstrates that the AGC mode reduces inhalational agent use without compromising haemodynamic stability or recovery time, making it superior to manual control. Furthermore, as emphasised in the literature, its safety and usage advantages encourage low-flow anaesthesia, with emphasis on its cost-effectiveness.

## Ethics

**Ethics Committee Approval:** This prospective, randomized controlled trial was conducted at Zonguldak Bülent Ecevit University Hospital between March 15, 2021, and March 10, 2022, after approval from the Zonguldak Bülent Ecevit University Ethics Committee (approval no.: 2021/05, date: 10.03.2021) and registration at ClinicalTrials.gov (NCT05404269).

**Informed Consent:** Written informed consent was obtained from all participants.

## Figures and Tables

**Table 1. Comparison of Demographic Characteristics and ASA Scores Between Groups:** 

-	**Group AGC** **(n = 30)**	**Group ManCo(n=30)**	**Group ModFA (n=30)**	***P***
**Age (years), median (IQR)**	49.5 (41.5-58.0)	52.5 (41.2-59.7)	53.0 (44.2-62.0)	0.517^a^
**Height (cm), mean ± SD**	161.9±5.9	160.7±5.1	162.2±5.2	0.519^b^
**Weight (kg), median (IQR)**	72.0 (62.0-80.0)	69.0 (65.0-73.8)	68.0 (56.8-79.5)	0.621^a^
**ASA I/II (n)**	6/24	14/16	7/23	0.050^c^

Data are presented as median (IQR) for non-normally distributed variables and mean ± SD for normally distributed variables.

ASA, American Society of Anesthesiologists; AGC, automatic gas control; ManCo, manual minimal-flow control; ModFA, manual medium-flow control; IQR, interquartile range; SD, standard deviation

^a^: Kruskal-Wallis test, ^b^: One-way ANOVA, ^c^: chi-square test.

**Table 2. Surgery-related Clinical Data:** 

-	**Group AGC** **(n = 30)**	**Group ManCo(n = 30)**	**Group ModFA(n = 30)**	***P* value**
**Surgical time (min), median (IQR)**	117.5 (97.5-135.0)	107.5 (95.0-130.0)	110.0 (96.3-125.0)	0.586^a^
**Anaesthesia time (min), median (IQR)**	132.5 (112.5-150.0)	125.0 (115.0-150.0)	125.0 (111.3-140.0)	0.651^a^
**Extubation time (min), mean ± SD**	14.0±1.2	19.4±3.1	14.1±1.4	**<0.001** ^b^
**Recovery time (min), median (IQR)**	2.5 (2.0-3.0)	3.0 (2.5-3.0)	2.5 (2.0-3.0)	0.097^a^

Data are expressed as median (IQR) or mean ± SD, as appropriate.

ASA, American Society of Anesthesiologists; AGC, automatic gas control; ManCo, manual minimal-flow control; ModFA, manual medium-flow control; IQR, interquartile range; SD, standard deviation; min, minimum

^a^: Kruskal-Wallis test, ^b^: One-way ANOVA.

**Table 3. Comparison of Sevoflurane Consumption and Cost Between Groups:** 

-	**Group AGC(n = 30)**	**Group ManCo(n = 30)**	**Group ModFA(n = 30)**	***P* value**
**Sevoflurane consumption (mL hour^-1^), mean ± SD**	7.01±1.10	9.80±1.54	15.05±1.28	**< 0.001** ^a^
**Sevoflurane cost (TL hour^-1^), median (IQR)**	24.6 (23.7-29.1)	35.6 (31.9-40.1)	55.7 (51.7-59.6)	**< 0.001** ^b^

Data are presented as mean ± standard deviation (SD) or median (interquartile range, IQR), as appropriate.

AGC, automatic gas control; ManCo, manual minimal-flow control; ModFA, manual medium-flow control; IQR, interquartile range; SD, standard deviation; TL, Turkish Lira

^a^: One-way ANOVA, ^b^: Kruskal-Wallis test.

**Table 4. Comparison of Time-point Sevoflurane Consumption Values Between Groups:** 

-	**Group AGC** ** (n = 30)**	**Group ManCo(n = 30)**	**Group ModFA(n = 30)**	***P* value**
**Sevoflurane consumption at 5 min (mL), median (IQR)**	1.5 (1.4-1.6)	2.4 (2.3-2.4)	2.4 (2.2-2.4)	< 0.001^a^
**Sevoflurane consumption at 10 min (mL), median (IQR)**	2.9 (2.7-3.2)	4.8 (4.7-4.8)	4.8 (4.6-5.0)	< 0.001^a^
**Sevoflurane consumption at 15 min (mL), median (IQR)**	3.8 (3.3-4.0)	7.2 (7.1-7.4)	6.8 (6.3-6.9)	< 0.001^a^
**Sevoflurane consumption at 20 min (mL), median (IQR)**	4.3 (4.0-4.9)	8.2 (7.9-9.1)	7.9 (7.5-8.4)	< 0.001^a^
**Sevoflurane consumption at 30 min (mL), median (IQR)**	5.5 (5.0-6.2)	9.3 (8.9-10.8)	10.4 (9.6-10.9)	< 0.001^a^
**Sevoflurane consumption at 60 min (mL), median (IQR)**	8.3 (7.8-9.5)	12.2 (11.8-14.1)	16.9 (15.6-18.3)	< 0.001^a^
**Sevoflurane consumption at 90 min (mL), median (IQR)**	11.2 (10.9-12.8)	15.4 (14.6-18.9)	24.5 (22.8-25.8)	< 0.001^a^
**Total sevoflurane consumption (mL), mean ± SD**	14.1±3.5	18.1±4.3	28.8±6.6	< 0.001^b^

Data are presented as median (IQR) or mean ± SD, as appropriate.

AGC, automatic gas control; ManCo, manual minimal-flow control; ModFA, manual medium-flow control; IQR, interquartile range; SD, standard deviation

^a^: Kruskal–Wallis test, ^b^: One-way ANOVA.

**Table 5. Comparison of Time-point BIS Values Between Groups:** 

-	**Group AGC** ** (n = 30)**	**Group ManCo(n = 30)**	**Group ModFA(n = 30)**	***P* value**
**At the 5^th^ minute after intubation (%), median (IQR)**	52.0 (49.0-60.0)	51.5 (48.2-55.5)	50.5 (48.0-54.0)	0.110
**At the 10^th^ minute after intubation (%), median (IQR)**	45.5 (42.0-51.5)	47.0 (41.0-50.8)	45.0 (40.0-48.8)	0.081
**At the 15^th^ minute after intubation (%), median (IQR)**	42.5 (40.2-49.5)	42.0 (40.2-46.8)	41.0 (39.0-44.0)	0.312
**At the 20^th^ minute after intubation (%), median (IQR)**	42.0 (40.0-48.0)	42.0 (40.2-46.0)	41.0 (39.0-45.0)	0.386
**At the 30^th^ minute after intubation (%), median (IQR)**	43.0 (41.2-46.0)	42.0 (40.0-45.5)	42.0 (40.0-44.8)	0.396
**At the 60^th^ minute after intubation (%), median (IQR)**	45.0 (41.2-48.0)	44.0 (41.2-46.0)	44.0 (41.2-49.0)	0.845
**At the 90^th^ minute after intubation (%), median (IQR)**	44.0 (41.0-47.0)	44.0 (41.0-50.0)	42.0 (40.0-48.0)	0.489
**Before extubation (%),median (IQR)**	88.0 (86.2-89.0)	88.0 (86.0-89.8)	88.0 (85.0-88.8)	0.336

Data are presented as median (IQR) as appropriate.

AGC, automatic gas control; ManCo, manual minimal-flow control; ModFA, manual medium-flow control; IQR, interquartile range
